# Domestic

**Published:** 1841-02-20

**Authors:** 


### DOMESTIC.

*Dr. Ticknor's Address to the Candidates for Degrees and Licenses, in the Medical Institution of Yale College, January* 20, 1841.—As a general rule, valedictory addresses to graduates are better than introductory lectures: they are often practical, sometimes eloquent; the one now before us belongs to the former class.

With much good advice, of a plain, sensible kind, Dr. Ticknor enjoins some pungent comments upon the conduct of a few of his medical brethren, sometimes too well merited.

"Again, let me invite your attention, for a moment, to those arts and tricks—the contemptible juggling and finessing—by which some men in the profession, as well as a vast multitude out of it, seek to forestall public opinion, and bespeak patronage and support. Among their artifices, are, reporting cases more or less dangerous than they are believed to be, calling them by new or unintelligible names, pretending to have discovered a new remedy or a new symptom, taking advantage of an alarmed patient or friends, a display of learning calculated to take with a certain class of people, and secrecy concerning the composition or cost of remedies, as though others did not possess them. Now, gentlemen, while these and a thousand similar arts are resorted to by men within the pale of medicine, neither the most learned discussions, nor the loudest denunciations against unprincipled pretenders to the healing art, can be expected to succeed. How contemptible in a physician, and a graduate too, to ride at the top of his speed, to throw himself into a sick room, out of breath, and with indications of profound learning and deep penetration, announce the astounding intelligence, 'that he is probably too late; but that, if there is any hope, any chance for the poor patient, he is the only man invested with adequate powers to meet the exigency.' Nothing should protect men, who resort to such means for self-advancement, or self-defence, from the charge of quackery: nay, more, it is piracy! Man is a sinning, and therefore a suffering and dying creature,—and theorize ever so wisely, and dream ever so long and sagely, our art will not always deprive death of his victims. It proposes to lessen human suffering, and prolong human life; not to perpetuate it."

Besides the mere trickery which is intended to advance personal interest, physicians are occasionally led from some good, or at least harmless motive, to take part in other pursuits or objects of general attraction. This is always of danger; the world is censorious in this respect, and it must be admitted that it has some reason in its rigorous inspection of the mental conduct of physicians. Medicine cannot be properly, still less efficiently pursued, without an unremitting direction of the intellect to it; and he who is evidently engrossed by other pursuits, is apt at last to think his profession an object of secondary importance. This does not, however, interfere with the proper culture of literature, or with that general acquaintance with men and things which add to the usefulness of a professional man. It is the disproportionate interest sometimes felt in them, which is apt to degenerate into abuse, that is censured, and properly enough.

"I consider it ominous of evil to all young men, setting out in life, to form a high relish for the light, dissipating literature of the day; but in a young physician, it is doubly unfortunate, if not even criminal, to spend late hours over the silly, catch-penny trash, at present so abundant, and yet so fascinating. Such a physician's fortune, I fancy, might be told, without reference to phrenological indications. All your time, all the energies of your mind, must be put in requisition, for all the resources of which you can acquire possession, will be wanted, as you advance in your professional career. If you succeed well in the practice of medicine, I suspect you will hardly find time and opportunity for much light reading, or any other fashionable mode of dissipation; nor even to make good your claims to political orthodoxy. When once public confidence is secured, a professional character established, and more especially when years of hard service begin to tell upon the constitution, physicians are strongly tempted to feel less interested in business matters, to manifest some reluctance to encounter hardships and privations, and so fall back upon their character for faithfulness and punctuality. This, the public will not bear with a very good grace, howsoever forbearing they may be towards men of other professions and pursuits. So, then, if you begin your career with prompt obedience to calls, and secure a firm standing by rendering your services with cheerfulness and apparent thankfulness; thus you must do, and thus you must do, so long as you make practice a business, or finally lose that confidence and standing."

---

*Weekly Report of Interments in the City and County of New York, from the 6th day of Feb. to the 13th day of Feb.*, 1841.—Diseases. Apoplexy 3; asthma 1; abscess 2; bleeding from lungs 1; burned or scalded 1; casualties 2; cancer 2; consumption 30; convulsions 14; croup or hives 2; diarrhœa 2; death from poison 1; dropsy 1; dropsy in the head 8; dropsy in the chest 1; drowned 1; dysentery 3; erysipelas 2; fever 3; do. scarlet 9; do. typhoid 3; do. puerperal 1; do. remittent 1; do. bilious 1; do. inflammatory 1; hip disease 1; intemperance 1; inflammation of throat 1; do. of womb 1; do. of brain 4; do. of liver 3; do. of chest 4; do. of lungs 14; do. of bowels 5; marasmus 6; measles 1; old age 1; organic disease of heart 1; rupture 1; small pox 3; scrofula 1; teething 2; unknown 2; whooping cough 1.

*Ages*—Of 1 year and under 41; between 1 and 2, 15; 2 and 5, 19; 5 and 10, 8; 10 and 20, 7; 20 and 30, 13; 30 and 40, 14; 40 and 50, 12; 50 and 60, 7; 60 and 70, 10; 70 and 80, 2; unknown, 1.

24 men—38 women—45 boys—42 girls. Total 149.

---

### HEALTH OF THE CITY.

INTERMENTS *in the City and Liberties of Philadelphia, from the 6th to the 13th of February,* 1841.

| Diseases. | Adults. | Children. |
|---|---|---|
| Apoplexy, | 1 | 0 |
| Asphyxia, | 0 | 1 |
| Croup, | 0 | 3 |
| Cong. of brain, | 3 | 0 |
| Childbed, | 1 | 0 |
| Consumption of the lungs, | 18 | 4 |
| Convulsions, | 1 | 3 |
| Diarrhœa, | 0 | 1 |
| Dropsy, | 1 | 0 |
| —— head, | 0 | 7 |
| Disease of the heart, | 1 | 0 |
| —— lungs, | 1 | 1 |
| Drowned, | 1 | 1 |
| Dysentery, | 2 | 0 |
| Debility, | 1 | 3 |
| Enlargement of heart, | 1 | 0 |
| Effusion on brain, | 0 | 1 |
| Fever, | 1 | 1 |
| —— scarlet, | 0 | 2 |
| Inflammation of the brain, | 0 | 1 |
| —— bronchi, | 0 | 2 |
| —— lungs, | 3 | 2 |
| —— stomach, | 1 | 0 |
| —— stomach and bowels, | 0 | 1 |
| —— bowels, | 0 | 1 |
| —— liver, | 1 | 1 |
| —— breast, | 0 | 1 |
| —— pleura, | 1 | 0 |
| *Carried forward*, | 39 | 37 |

| Diseases. | Adults. | Children. |
|---|---|---|
| *Brought forward*, | 39 | 37 |
| Infl. of throat, | 0 | 1 |
| —— peritoneum, | 0 | 1 |
| Intemperance, | 1 | 0 |
| Marasmus, | 1 | 2 |
| Nervous irritation, | 0 | 1 |
| Old age, | 3 | 0 |
| Palsy, | 1 | 0 |
| Rheumatism, | 1 | 0 |
| Small pox, | 0 | 1 |
| Still-born, | 0 | 7 |
| Suicide, | 1 | 0 |
| Suffocation, | 1 | 0 |
| Unknown, | 0 | 1 |
| Vomiting of blood, | 0 | 1 |
| Total, 100— | 48 | 52 |

Of the above, there were under 1 year, 25

| | |
|---|---|
| From 1 to 2 | 7 |
| 2 to 5 | 13 |
| 5 to 10 | 3 |
| 10 to 15 | 2 |
| 15 to 20 | 2 |
| 20 to 30 | 12 |
| 30 to 40 | 9 |
| 40 to 50 | 6 |
| 50 to 60 | 7 |
| 60 to 70 | 5 |
| 70 to 80 | 7 |
| 80 to 90 | 2 |
| 90 to 100 | 0 |
| Total, | 100 |

In the above are included 15 people of colour, and 12 interments from the alms-house.

---

### MEETING OF THE KAPPA LAMBDA SOCIETY, NEW YORK.

*Dislocation of the Os Femoris in an Adult Female.*—Dr. Rodgers mentioned that he had seen another case of dislocation of the os femoris in an adult female. He has already reported three cases of this accident, which have come under his care, and which, so far as he can learn, is of extremely rare occurrence. This case, which makes the fifth case he has seen, and the fourth which he has reduced, the other case being irreducible, occurred two months since, in a woman eight months advanced in pregnancy, who was thrown from a wagon. It presented the ordinary symptoms of dislocation, with the head of the bone resting on the dorsum of the ileum, and was reduced without trouble.

To show the rarity of this accident, we may mention that at the suggestion of Prof. Warren, of Boston, we have for some years been in the habit of inquiring of surgeons both in Europe and America, the number of instances with which they had met. We have only once encountered any one who could personally recall a case.

*Remarkable effects of Rheumatism.*—Dr. Washington mentioned an instance of the remarkable effects of rheumatism which he had witnessed in a young man nineteen years of age, brother of one of the teachers of the Deaf and Dumb Asylum. He has been suffering from it ten years, and lost a brother who had it the same length of time. His knees are dislocated so that the condyles of the femur are anterior to the head of the tibia; the bones of the spine are anchylosed; the chin is fixed within two fingers' breadth of the top of the sternum, so that he cannot move his head without moving the whole body; the union of the bony surfaces is apparently perfect. The sight of the right eye has been destroyed by iritis, and the pupil of the other eye is so contracted by the same disease, as to prevent his reading. There is great emaciation of the whole system, and particularly of the limbs, so that his thigh is not larger than the wrist of an ordinary sized man. There is now active disease in both wrists, which are tender, and have a doughy feel. His digestive organs are in good order, his tongue slightly furred. He is of a cheerful disposition, and continued to read as long as the state of his eyes permitted. Dr. W. never saw any thing approaching such a degree of deformity.

*Case of probable sloughing away of the Uterus.*—Dr. A. C. Post mentioned the probable occurrence of this in the case of a woman whom he had seen, twenty-eight years of age, who was married three years since, and had two abortions within two years. The last one occurred in May last, during two months after which time, she stated that she was more or less unwell, though able to be about the house. She suffered from a sensation of dragging and pain in the lower part of the abdomen, accompanied, as she said, with an unusual protrusion, which must have been either inversion or prolapsus of the uterus, and which, she said, came away with a fœtid smell. On a recent examination, Dr. P. found a complete closure of the vagina half an inch beyond the meatus urinarius, which was so firm that the resistance could not be overcome. There was an indurated cicatrix between the vagina and the rectum. Dr. P. thinks that the uterus must have sloughed away.—*New York Journ. of Med.*

